# Influence of propofol on isolated neonatal rat carotid body glomus cell response to hypoxia and hypercapnia

**DOI:** 10.1016/j.resp.2018.10.007

**Published:** 2019-02

**Authors:** Peadar B. O'Donohoe, Philip J. Turner, Nicky Huskens, Keith J. Buckler, Jaideep J. Pandit

**Affiliations:** a Department of Physiology, Anatomy & Genetics, Parks Road, University of Oxford, Oxford, OX1 3PT, UK; b Nuffield Department of Anaesthetics, Oxford University Hospitals NHS Trust, Oxford, OX3 9DU, UK

**Keywords:** Carotid body, Chemosensitivity, Hypoxia, Propofol

## Abstract

In humans the intravenous anaesthetic propofol depresses ventilatory responses to hypoxia and CO_2_. Animal studies suggest that this may in part be due to inhibition of synaptic transmission between chemoreceptor glomus cells of the carotid body and the afferent carotid sinus nerve. It is however unknown if propofol can also act directly on the glomus cell. Here we report that propofol can indeed inhibit intracellular Ca^2+^ responses to hypoxia and hypercapnia in isolated rat glomus cells. Neither this propofol effect, nor the glomus cell response to hypoxia in the absence of propofol, were influenced by GABA receptor activation (using GABA, muscimol and baclofen) or inhibition (using bicuculline and 5-aminovaleric acid). Suggesting that these effects of propofol are not mediated through GABA receptors. Propofol inhibited calcium responses to nicotine in glomus cells but the nicotinic antagonists vecuronium and methyllycaconitine did not inhibit calcium responses to hypoxia. TASK channel activity was not altered by propofol. The glomus cell Ca^2+^ response to depolarisation with 30 mM K^+^ was however modestly inhibited by propofol. In summary we conclude that propofol does have a direct effect upon hypoxia signalling in isolated type-1 cells and that this may be partially due to its ability to inhibit voltage gated Ca^2+^_v_ channels. We also note that propofol has the capacity to supress glomus cell excitation via nicotinic receptors and may therefore also interfere with paracrine/autocrine cholinergic signalling in the intact organ. The effects of propofol on chemoreceptor function are however clearly complex and require further investigation.

## Highlights

• The intravenous anaesthetic propofol acts directly on carotid body glomus cells to inhibit their response to hypoxia.
• Propofol acts via novel mechanisms, as we excluded action via its known target receptors (nicotinic, GABA-ergic, or K+ channel).
• Inhibition of the hypoxic response is clinically relevant in anaesthesia.

## 1 Introduction

In humans the anaesthetic propofol inhibits ventilatory response to both hypoxia and (to a lesser extent) to hypercapnia (Blouin et al., 1993; Nagyova et al., 1995). These effects are similar to that seen with many volatile anaesthetic agents (Knill and Gelb, 1978; Dahan et al., 1994; van den Elsen et al., 1995; Sollevi and Lindahl, 1995; Pandit et al., 1999a,b; Pandit, 2002, 2005). Whilst the full clinical implications of these observations are unclear a robust response to hypoxia and CO_2_ is clearly beneficial during recovery from surgery and even modest inhibition of respiratory chemoreflexes can influence outcomes (Knill and Gelb, 1982). These observations have stimulated research into the underlying mechanisms by which general anaesthetics influence chemoreflexes.

Much of this research has focussed upon the carotid bodies as these seem to be directly sensitive to many general anaesthetics. For example Jonsson, et al. (2005) demonstrated that propofol inhibited nerve excitation by hypoxia in an isolated rabbit carotid body preparation. Within these organs there are a number of potential targets for the actions of anaesthetic. The most likely sites of action are at the type-1 or glomus cell, wherein hypoxia and acidosis are sensed, and at the afferent nerve ending. In this paper we have focussed our attention upon the possible effects of propofol on sensory transduction in the glomus cell since this seems to be a major target for the action of volatile anaesthetics (Buckler et al., 2000; Pandit and Buckler, 2009).

Glomus cells respond to hypoxia, hypercapnia and acidosis with an increase in intracellular Ca^2+^([Ca^2+^]_i_) which promotes vesicular release of various neurotransmitters including ATP, dopamine and acetylcholine, activating the postsynaptic (afferent nerve terminal) membrane (Buckler and Vaughan-Jones, 1994a, b; Dasso et al., 2000; Toledo-Aral et al., 2002; Fearon et al., 2003a, 2003b; Zhang et al., 2012; Fitzgerald et al., 1999; Shirahata et al., 2007). Postsynaptic excitation then initiates action potential generation in the carotid sinus nerve which signals to the breathing centres of the brainstem (Weir et al., 2005). The initial mechanisms of oxygen (O_2_) sensing are complex and contentious but there is a consensus that both acidosis and hypoxia excite the type-1 cell through the inhibition of potassium channels. Of particular relevance is the inhibition of TASK channels which normally maintain the glomus cells resting membrane potential since this leads inevitably to membrane depolarisation and the initiation of voltage gated calcium entry (Buckler, 2015). In addition the glomus cell is highly regulated by neurocrine, autocrine and paracrine mechanisms (Nurse, 2014). For example GABA is thought to provide presynaptic autoregulatory feedback during hypoxia (Fearon et al., 2003b) and adenosine causes presynaptic autostimulation (Vandier et al., 1999).

In principle anaesthetics could act upon glomus cells at any point within this transduction cascade and its associated paracrine/autocrine regulatory pathways. For volatile anesthetics, the leading candidate mediators for the depressive effects on hypoxia/CO_2_ response are the TASK channels which are activated by halothane, isoflurane and sevoflurane (Buckler et al., 2000; Pandit et al., 2010a). Propofol may however have different actions since it has little effect upon cloned TASK-1 channels (Putzke et al., 2007). Jonsson et al. (2005) found that propofol not only suppressed the excitatory effects of hypoxia but also those of nicotine suggesting a possible action upon cholinergic signalling. This idea is consistent with observations that curare-like neuromuscular blocking drugs also inhibit the hypoxic response (Eriksson, 1999; Jonsson et al., 2004), that nicotinic receptors are present in glomus cells (Dasso et al., 1997) and that nicotinic receptors may be involved in mediating the hypnotic actions of propofol (Flood et al., 1997; Violet et al., 1997; Tassonyi et al., 2002).

Hitherto however, the effects of propofol on chemoreflex drive have been studied only in humans (Blouin et al., 1993; Nagyova et al., 1995), isolated perfused carotid body preparations (Jonsson et al., 2004, 2005; Akada et al., 2008) or intact, anesthetised animals (Ponte and Sadler, 1989). Possible direct actions upon glomus cells have not been investigated. Here we close that gap and present the results of a study into the effects of propofol on hypoxia sensing in isolated type-1 cells.

## 2 Material and methods

All animal procedures were performed in accordance with project and personal licence authorities issued under the UK Animals (Scientific Procedures) Act, 1986.

### 2.1 Cell isolation

Carotid bifurcations were dissected *in situ* from neonatal Sprague-Dawley rats (P11-14) under terminal isoflurane anesthesia (4% isoflurane in oxygen) and placed in ice-cold saline. Animals were supplied by Harlan (Blackthorn, Oxfordshire, UK). Microdissection of the carotid body was carried out *ex vivo*, and after enzymatic treatment the tissue was triturated to isolate individual cells in suspension as previously described (O’Donohoe et al., 2018). Primary cell cultures in DMEM/F12 (supplemented with 10% foetal bovine serum, glutamine 2 mM, insulin 8 μg/ml, penicillin 100 units/ml and streptomycin 100 μg/ml) were plated onto coverslips and incubated (37 °C with 5% CO_2_ in air) for two hours prior to loading with 2.5μM indo-1-AM for 60 min.

### 2.2 Calcium imaging

An inverted microscope (Nikon Diaphot) with 40x oil-immersion objective was used to identify glomus cells, which were excited at a wavelength of 340 nm by filtered light from an Xenon arc lamp. In epifluorescence mode, emitted indo-1 fluorescence was split and measured at 405 ± 10 nm and 495 ± 10 nm by two trialkali photomultiplier tubes (Thorn EMI). Intracellular calcium concentration ([Ca^2+^]_i_)was two-point calibrated *in situ* with a separate group of cells rendered calcium permeant with 5μM ionomycin then perfused with 100μM EGTA (0 mM Ca^2+^) and 10 mM CaCl_2_ (both in a 10 mM HEPES buffered high K^+^ saline containing 140 mM KCl, pH 7.4) to determine R_min_, R_max_ and F495_free/bound_. The calibrated ratio of the intensities was then used to estimate [Ca^2+^]_i_ in our cells of interest using the following equation:

$${\left[{Ca}^{2+}\right]}_{i}\;=K_{d}+\left(F_{495,\frac{free}{bound}}\right).\frac{R-R_{min}}{R_{max}-R}$$

Where R is the measured ratio of emission at 405/495 nm, K_d_ is the dissociation constant for indo-1 AM dye from Ca^2+^ (250 nM), F495_free/bound_ the ratio fluorescence intensity of free indo-1 to Ca^2+^-bound indo-1 at 495 nM, R_min_ is the 405/495 nm fluorescence ratio of unbound indo-1 and R_max_ the ratio of Ca^2+^-bound indo-1 (Buckler and Vaughan-Jones, 1993).

### 2.3 Perfusion system

Warmed gas equilibrated Tyrode solutions (see below) were passed through a small glass bottomed chamber (volume ∼100 μl) at a flow rate of 5–8 ml/min. A two way tap was used to change the fluid flowing through the chamber with a complete volume change occurring in less than 5 s (Huskens et al., 2016). Temperature was maintained at 37 °C using a heating element immediately upstream of the chamber.

### 2.4 Hypoxia experiments

Cells were superfused with 37 °C Tyrode’s solution containing, in mM, 117 NaCl, 2.5 CaCl_2_, 4.5 KCl, 1 MgCl_2_, 23 NaHCO_3_, 11 glucose) equilibrated with 5% CO_2_ in air (euoxia) or 5% CO_2_ in N_2_ (hypoxia). Hypoxia resulted in a PO_2_ of ∼ 3 mmHg in the perfusion chamber (measured using a fluorescence quenching optode; Huskens et al., 2016). This was designed to elicit robust activation of cells.

### 2.5 Isohydric hypercapnia experiments

Cells were initially superfused with euoxic solutions containing 5% CO_2_ (pH7.4), as previously described, to mimic typical physiological CO_2_ tensions. Hypercapnic Tyrode’s solution was equilibrated with 20% CO_2_ in 80% air. Hypercapnic solutions also contained elevated levels of NaHCO_3_ (substituted for the same molar amount of NaCl) so as to maintain a constant pH of 7.4 at 37 °C (isohydric hypercapnia; Zhang and Nurse, 2004), as measured directly by a pH meter.

### 2.6 High [K^+^]o experiments

Each cell was challenged with the standard hypoxic stimulus as a positive control for identifying functional glomus cells, prior to superfusion with a Tyrode’s solution modified to contain 30 mM KCl (with equimolar reduction in NaCl). This depolarises the plasma membrane initiating voltage gated Ca^2+^entry. At this level of [K^+^]_o_, the Nernst equation predicts the membrane to depolarize to approximately-41 mV (i.e. the calculated equilibrium potential for K^+^, assuming that [K^+^]_i_ is 140 mM). Under these depolarized conditions, activation of voltage-gated Ca^2+^ channels causes an increase in [Ca^2+^]_i_ in the glomus cell (Buckler and Vaughan-Jones, 1994a; Overholt and Prabhakar, 1997).

### 2.7 Electrophysiology experiments

Cell-attached patch clamp electrophysiology was performed using an Axopatch 200B and pipettes made from borosilicate glass capillaries coated with Sylgard (Dow Corning, Seneffe, Belgium). Pipettes were fire-polished immediately before filling. Cell-attached filling solution contained 140 mM KCl, 1 mM MgCl_2_, 1 mM EGTA,10 mM HEPES, 10 mM tetraethylammonium (TEA) and 5 mM 4-aminopyridine (4-AP) at pH 7.4 at 37 °C. Recordings were conducted in a high K^+^ modified Tyrode’s solution (containing in mM: 100 KCl, 21.5 NaCl, 23 NaHCO_3_, 11 glucose), to depolarise and therefore stabilise the cell membrane potential. Once a seal had been formed in normal Tyrode the patch was held at a pipette potential of +80 mV and the cell perfused with the above high K ^+^ Tyrode. This results in a predicted membrane potential for the patch of about – 90 mV. Membrane current was filtered at 2 kHz and digitized at 20 kHz prior to data acquisition with Spike2 software. Under these recording conditions (i.e. at negative membrane potentials of -60 to -90 mV and with TEA and 4-AP in the pipette solution), single channel activity is predominantly due to heterodimers of TASK-1/TASK-3 with some homodimeric TASK-1 and TASK-3. This is based upon biophysical evidence (Williams & Buckler 2004; Kim et al., 2009), *Task1* and *Task3* gene disruption (Turner and Buckler, 2013) and pharmacological evidence (O’Donohoe et al., 2018). The main conductance state (TASK-1/TASK-3) for each recording was defined using an all points histogram, and the threshold for opening set at 50% of this value. Multiple openings were defined as current of 150%, 250%, 350%, etc., of the main conductance state, as multiple channels were often present in a patch. Channel activity was thus quantified as NP_open_. Measurements of NP_open_ were performed on 20 s sections of recording made before and during application of propofol.

### 2.8 Drugs

All drugs were appropriately reconstituted daily prior to dissolution in Tyrode’s solution. Pure 2,6-di-isopropylphenol was found immiscible in Tyrode; propofol-DMSO resulted in a visible precipitate when the Tyrode solution was bubbled with gas, and we wished to avoid the use of ethanol as a solvent (Fourcade et al., 2004) as it may have independent anaesthetic effects (Garfield and Bukusoglu, 1996)· Consequently we used propofol dissolved in 10% Intralipid (Fresenius, Runcorn, Cheshire, UK) to prepare Tyrode solutions containing 1–500 μM propofol. We investigated and excluded any possible direct effects of Intralipid on the hypoxic response (see results), as have others (Jonsson et al., 2005). Another potential concern is whether propofol is retained in the Intralipid phase rather than free in Tyrode solution. Reassuringly Kalitynski et al. (2006) addressed this problem using high performance gas liquid chromatography and reported no significant influence of Intralipid on free concentrations of propofol > 3 μM in Tyrode, or Tyrode plus albumin or human plasma. It was only at very low propofol concentrations < 3 μM that free propofol levels were significantly lower with Intralipid. We therefore believe the stated concentrations of propofol used in this study to represent that of free propofol.

GABA (Sigma-Aldrich, Gillingham, Dorset, UK) was applied in concentrations of 5 μM as a synaptic concentration to restore tonic GABA activity or 1 mM to evoke maximal GABA activity. Muscimol 50μM (Sigma-Aldrich) was used to selectively activate GABA_A_, which was in turn antagonised to exclude tonic activity by bicuculline 100 μM. Baclofen 50 μM (Abcam, Cambridge, Cambridgeshire, UK) was used to activate and 5-aminovalleric acid 100 μM (5-AVA, Sigma-Aldrich) to inhibit GABA_B_ in isolation. Nicotine 300 μM (Sigma-Aldrich) was used as a selective nicotinic acetylcholine receptor (nAChR) agonist, vecuronium 10 μM (Abcam) as a competitive, non-selective antagonist of nAChR, while methyllycaconitine 50 μM (MLA, Abcam) was used as a selective nAChR antagonist to ensure blockade of all (including α7 homomeric) nAChRs if no effect of vecuronium was observed.

Choice of dosing of propofol was guided by the results we obtained. First, our study of the effects of propofol on hypoxic response over a very wide range of concentrations (10–500 μM) yielded a ‘dose-response relationship’ for its effect on the primary variable of interest. To compare this with the effects of propofol on CO_2_ sensing, we therefore planned to use a dose of propofol at approximately mid-point of this hypoxic dose-response relationship, so that we could more readily assess if the effect of propofol on CO_2_ sensing was greater or lesser than on O_2_ sensing. GABA putatively potentiates the action of propofol (since propofol as described above is purported act via GABA receptors) and hence we planned to use a very modest propofol dose that itself little influenced hypoxic response, so that any augmentation by GABA would be evident. It would have been fruitless to use a high dose of propofol that already near-maximally depressed hypoxic response, as this would then mask any additional effect of GABA. Conversely, nicotine is known to evoke a rise in [Ca^2+^]_i_ and so we planned to employ a propofol concentration that had a substantial but non-saturating effect on depressing hypoxic response. This would allow us to compare the relative effects of propofol on the two stimuli (hypoxia and nicotine).

### 2.9 Statistics

For any single data point for each condition within each protocol we aimed to obtain the very minimum of at least 4 recordings of [Ca^2+^]_i_ or channel activity from different cells. In most cases however we obtained much larger datasets (especially in protocols concerning dose-response relationships) in order to maximise the overall power of the study. Data are presented in mean ± SEM unless otherwise stated. Comparisons of two groups were analysed using Student’s *t*-test (paired tests for control vs test as these experiments were conducted on the same cells). Where more than two groups were compared, ANOVA was used with post hoc tests with adjustment (Bonferroni) for multiple testing, as appropriate (Pandit, 2010). In the ANOVA, the ‘response’ was [Ca^2+^]_i_ effect (ie, the ratio of test vs control) and the ‘factors’ were (where relevant) either drug dose (one level for each dose) or drug (where for example, drug vs no drug conditions were being compared, or where drug vs drug + antagonist were being compared, with one level for each such condition). Calculations were performed using SPSS (Version 20) for Windows (IBM Corp, Armonk, NY, USA).

## 3 Results

### 3.1 Effect of propofol on glomus cell response to hypoxia

**Fig. 1:**
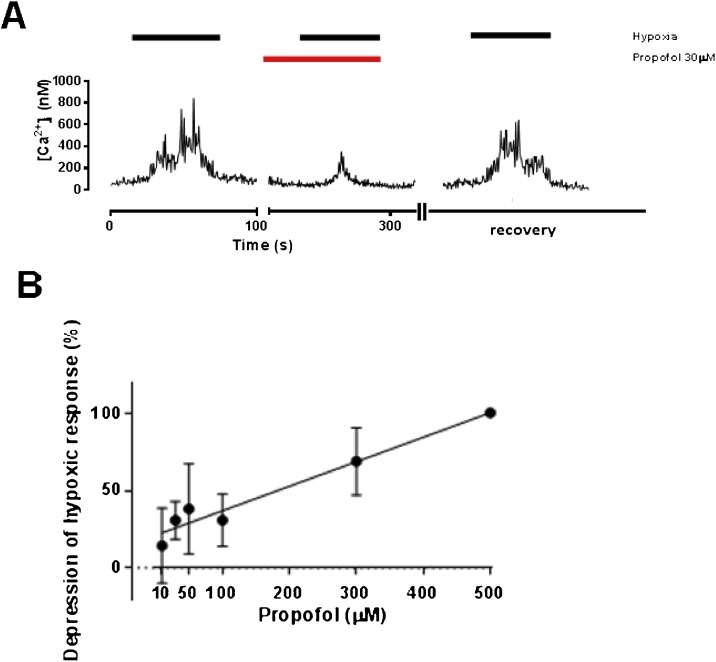
Influence of propofol on hypoxia-induced [Ca^2+^]_i_ response in isolated glomus cells. Example trace of [Ca^2+^]_i_with exposures to hypoxia, showing the inhibition of hypoxic response in this cell by 30 μM propofol. B. concentration-response relationship of propofol on hypoxic response of glomus cell (magnitude of depression of Ca^2+^ transient) with propofol dose (μM; mean ± SEM, each point average of 4–8 recordings, 30 in total. At 1–2 μM propofol the effect was negligible (data not shown).

Propofol caused a significant dose-dependent depression of the hypoxia-induced rise in [Ca^2+^]_i_ (Fig. 1A-B; p = 0.043 for dose, ANOVA), and at a dose of 30 μM (corresponding to a clinical concentration of ∼5 μg/ml; Jonsson et al., 2005) reduced the response from 437 ± 133 nM to 255 ± 153 nM (n = 9; p = 0.015). At lower concentrations (1–2 μM) propofol had no discernible effect on hypoxic response. The clinical relevance of these concentrations is considered in the discussion. In control experiments Intralipid 10% into which propofol is suspended in its clinical presentation, had no effect alone on hypoxia induced rise in [Ca^2+^]_i_ (183 ± 48 vs 159 ± 63 nM; n = 11, p = 0.469).

### 3.2 Effect of propofol on glomus cell response to CO_2_

**Fig. 2:**
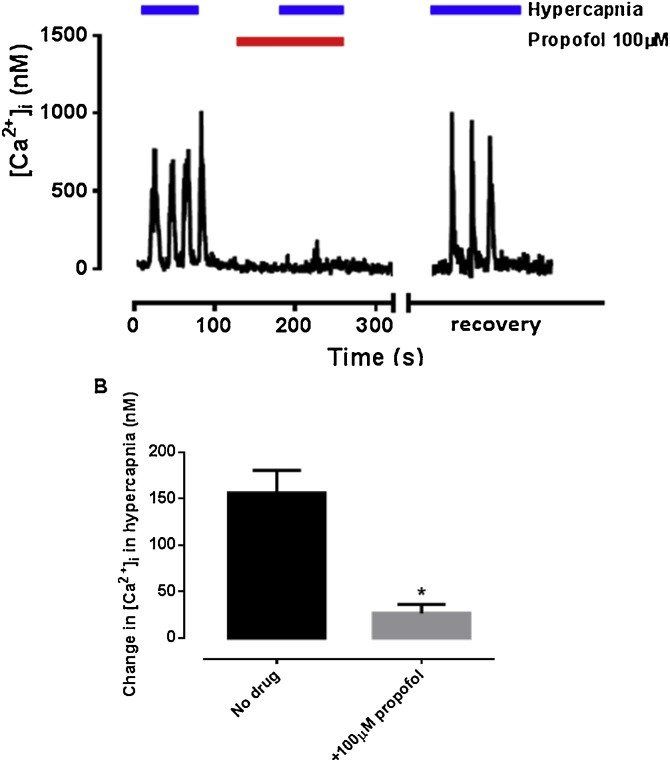
A. Influence of propofol on 20% CO_2_-induced [Ca^2+^]_i_ response in isolated glomus cells. The cell was first assessed for oxygen sensitivity (note response to hypoxia). Then 20% CO_2_was introduced as the stimulus (hypercapnia). This response was near-abolished by 100 μM propofol; an effect which was reversible after washout. B. Quantitative analysis of the response (mean ± SEM; n = 7). * represents significance at p < 0.05.

**Fig. 3:**
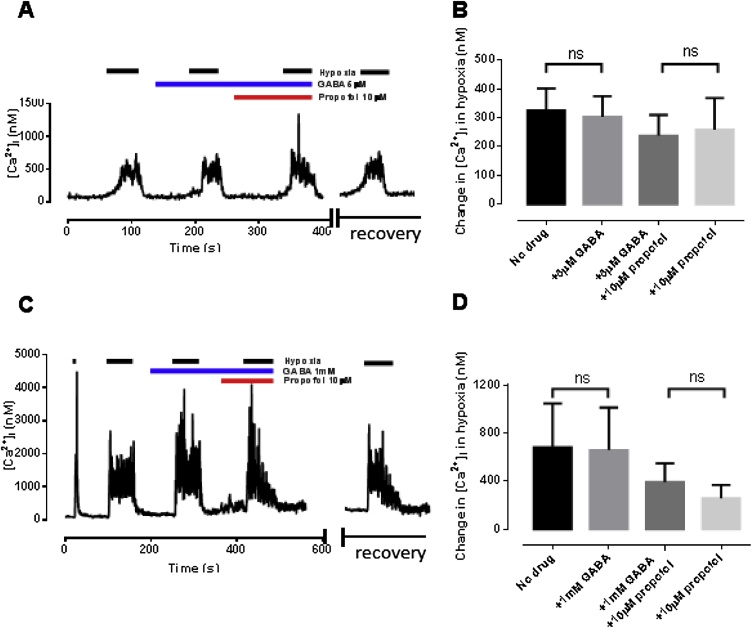
Perfusion of glomus cells with GABA alone (5 μM in panel A; 1 mM in panel B) did not influence the [Ca^2+^]_i_ response to hypoxia. Furthermore, the addition of GABA had no additional influence on the effect of propofol at a concentration of 10 μM (panels B and D; n = 5–8).This suggests that GABA has no effect across a wide concentration range and that the effect of propofol on O_2_ sensing is not influenced by GABA.

Fig. 2 shows the effect of propofol on the intracellular [Ca^2+^]_i_ response to 20% CO_2_. In this figure it is evident that, in the absence of propofol, intracellular [Ca^2+^]_i_ shows a repetitive ‘spiking’ pattern of response to hypercapnia which is representative of a typical glomus cell response to this stimulus (Buckler and Vaughan-Jones, 1993; Pandit et al., 2010b). By consistently quantifying the [Ca^2+^]_i_ response as an average over the first minute of exposure, we accounted for this spiking behaviour and also avoided any confounding influence on our estimates of the later decline in [Ca^2+^]_i_ that is sometimes observed with sustained hypercapnia (Buckler and Vaughan-Jones, 1994a, b; Dasso et al., 2000; Pandit et al., 2010b). The effect of propofol was assessed at one dose only, 100μM which had yielded a clear depressive effect on the hypoxic response (Fig. 1). Thus 100 μM propofol caused a large decrease in the [Ca^2+^]_i_ response to 20% CO_2_ from 156 ± 24 nM to 27 ± 10 nM (n = 7, p = 0.003; Fig. 3).

The fact that propofol exerts similar effects upon the glomus cell response to both hypoxia and acidosis suggests that its actions may lie along a final common pathway shared by both stimuli; i.e., between ion/potassium channel regulation, calcium signalling, and neuromodulation by autocrine signalling pathways. Since propofol is known to interact with both GABA_A_ receptors and nicotinic cholinergic pathways we first investigated the role of these in chemoreceptor function.

### 3.3 Effect of GABA modulation on glomus cell response to hypoxia

Perfusion of glomus cells with GABA alone across a very wide, 200-fold dose range (5 μM to 1 mM; Fig. 3A and B) did not influence either the baseline euoxic [Ca^2+^]_i_ or the [Ca^2+^]_i_ response to hypoxia (Fig. 3; p = 0.813 and p = 0.136 for 5 μM and 1 mM GABA respectively). Furthermore, propofol at a dose of 10 μM (which alone depressed hypoxic response by just 14%, Fig. 1B) was studied in the presence of either 5 μM or 1 mM GABA. In statistical testing the interactive term of the factors ‘propofol’ and ‘GABA’ had no statistically significant effect (ANOVA, F = 0.298, p = 0.589; F = 0.385, p = 0.541 respectively), confirming no synergistic effect of GABA with propofol.

**Fig. 4:**
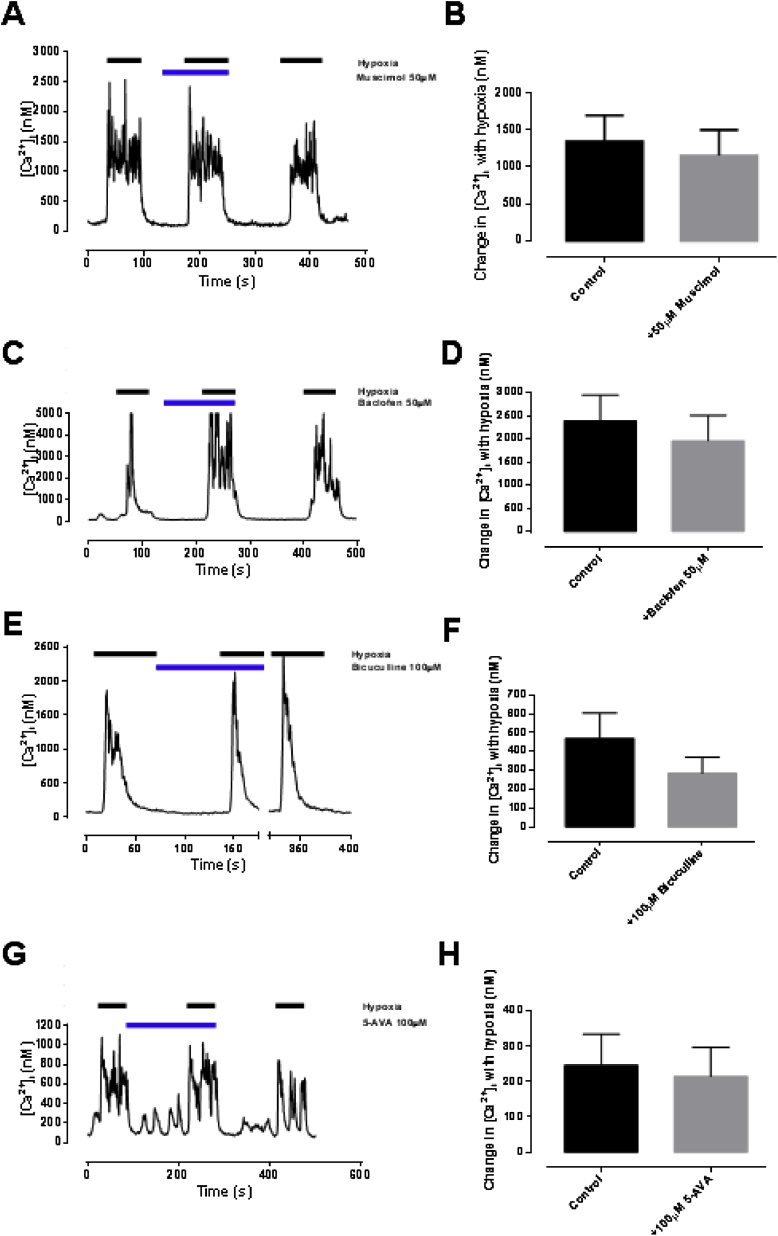
Null effect of specific GABA agonists and antagonists on hypoxic response (see text). A-B muscimol (GABA-A agonist; n = 8) C-D baclofen (GAB A-B agonist; n = 7) E-F bicuculline (GABA-A antagonist; n = 7) and G-H. 5-AVA (GAB A-B antagonist; n = 17). All figures [Ca^2+^]_i_ in nM ± SEM.

To assess for specific effects of GABA_A_ vs GABA_B_ agonism, selective subtype agonists and antagonists were also studied. None of the following had any significant effect on either baseline euoxic [Ca^2+^]_i_ or the magnitude of [Ca^2+^]_i_ response to hypoxia (Table 1): muscimol (a GABA_A_ agonist; Krogsgaard-Larsen and Johnston, 1978) 50 μM; baclofen, (a GABA_B_ agonist; Hill and Bowery, 1981) 50 μM; bicuculline (a GABA_A_ antagonist; Macdonald et al., 1989) 100 μM; 5-AVA (a GABA_B_ antagonist; Muhyaddin et al.,1982) 100 μM; Fig. 4. Because of the complete lack of effect of GABA (Zhang et al., 2009) and of GABA antagonists/agonists on the [Ca^2+^]_i_ response to hypoxia interaction these selective agonists/antagonists with added propofol was not investigated.

**Table 1:** Effect of GABA modulators on euoxic baseline and hypoxia-induced increase in [Ca^2+^]_i_. All figures [Ca^2+^]_i_ in nM ± SEM. Note the wide variation in responsiveness of batches of cells which underlines the importance of paired interventions.

	Euoxic baseline [Ca^2+^]_i_			Hypoxia induced increase in [Ca^2+^]_i_		
	No drug	Drug	p	No drug	Drug	p
Muscimol (n = 8)	162 ± 32	128 ± 20	0.129	1350 ± 349	1152 ± 351	0.238
Baclofen (n = 7)	198 ± 40	143 ± 18	0.262	2385 ± 564	1959 ± 550	0.427
Bicuculline (n = 7)	181 ± 38	165 ± 37	0.721	469 ± 134	285 ± 84	0.274
5-AVA (n = 17)	148 ± 24	120 ± 20	0.220	247 ± 88	215 ± 82	0.664

### 3.4 Effect of propofol on nicotinergic stimulation of glomus cells

**Fig. 5:**
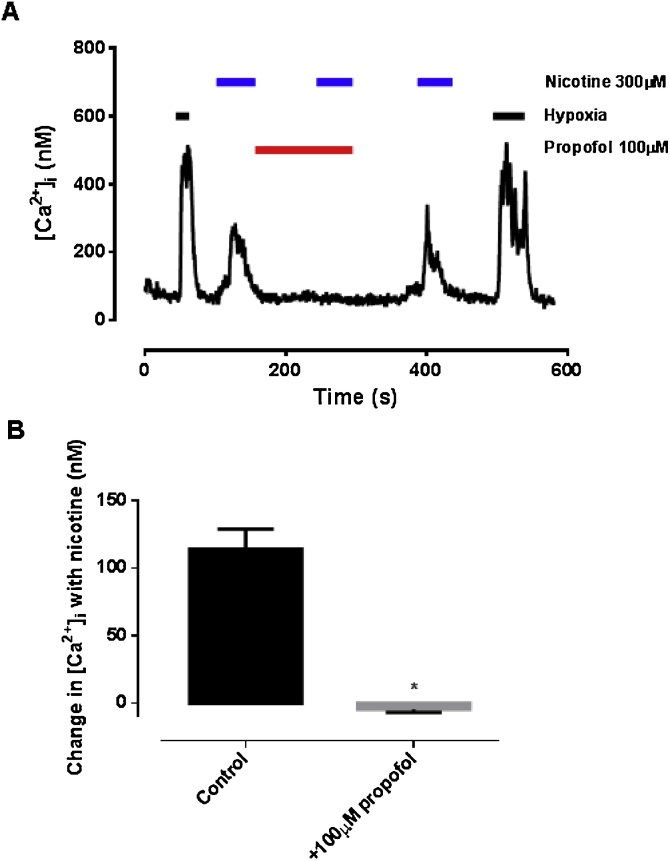
A. Effects of nicotine on [Ca^2+^]i in isolated glomus cells. Note the initial rise in [Ca^2+^]i with nicotine, its near abolition by the nicotinic cholinergic antagonism vecuronium, and restoration of nicotine-induced [Ca^2+^]i response after washout of vecuronium. B. Quantitative analysis (mean ± SEM; n = 7, p < 0.001).

**Fig. 6:**
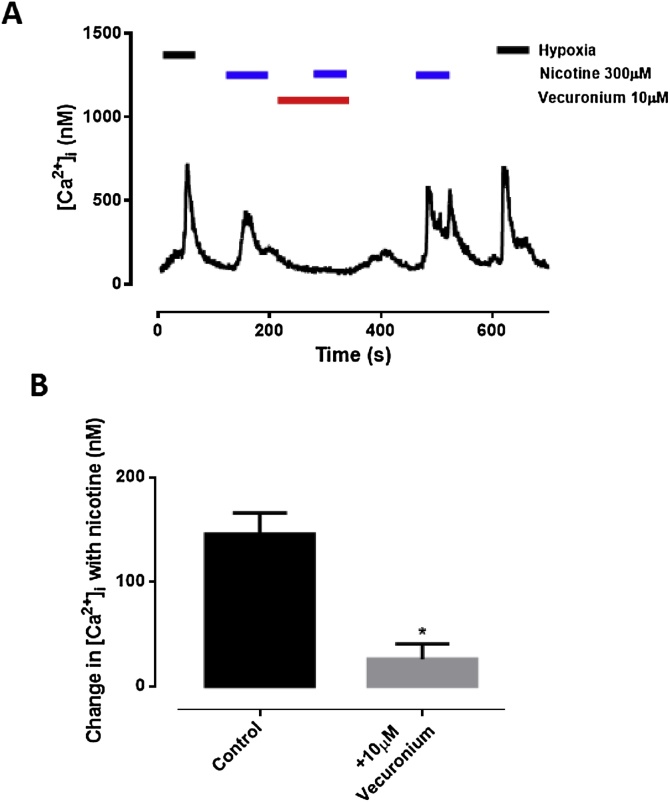
A. Representative trace of vecuronium influence on nicotine-induced rise in [Ca^2+^]i in isolated glomus cells. Note the initial rise in [Ca^2+^]i with nicotine, its near-abolition by vecuronium, and restoration of nicotine-induced [Ca^2+^]i response after washout. B. Quantitative analysis (mean ± SEM; n = 5, p = 0.015).

**Fig. 7:**
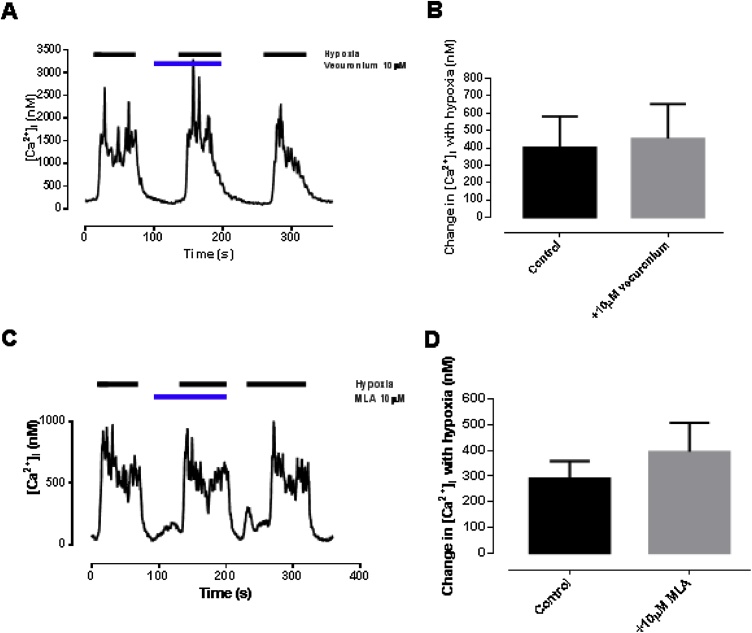
A-B. Vecuronium has no effect on hypoxia induced increase in [Ca^2+^]_i_ (n = 6; p = 0.5). C-D. MLA showed no effect on hypoxia induced increase in [Ca^2+^]_i_ (n = 5, p = 0.367) All figures mean ± SEM.

Fig. 5 shows the effects of nicotine (300 μM) on intracellular calcium in isolated type-1 cells. As previously reported (Dasso et al., 1997) nicotine caused a rapid and robust increase in intracellular calcium which was substantially inhibited by vecuronium (Fig. 6; p = 0.015). Propofol (100 μM) also substantially inhibited the effects of nicotine (Fig. 5B; p < 0.001). Unlike propofol however, neither vercuronium nor MLA influenced the *hypoxia-induced* increase in [Ca^2+^]_i_ (Fig. 7; p = 0.807). Thus whilst nicotinic receptors are present in type-1 cells and can be antagonised by propofol this process does not appear to play a significant role in mediating the effects of propofol on chemosensing in isolated type-1 cells. We therefore turned our attention to processes upstream of neuromodulation.

### 3.5 Effect of propofol on TASK channels

**Fig. 8:**
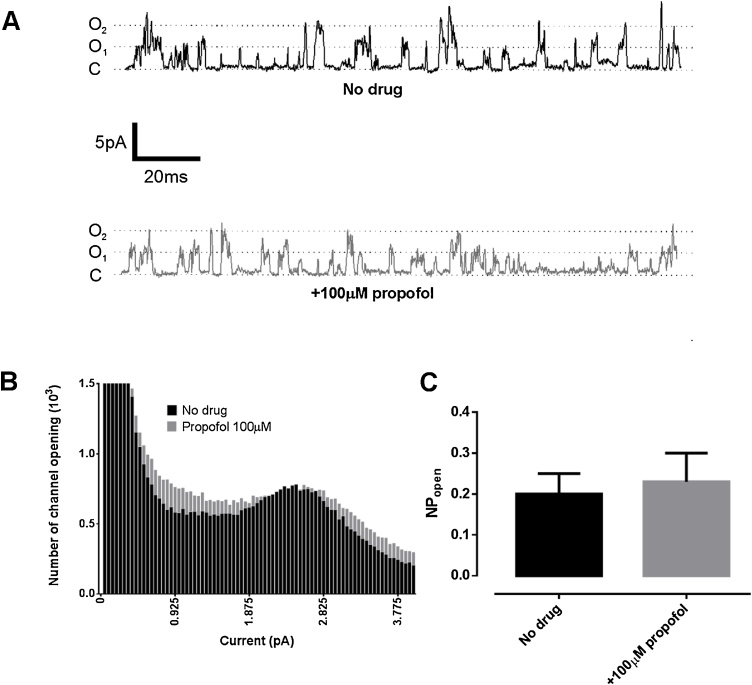
Cell attached patch recording of TASK channel activity in a type-1 cell. Pipette potential + 80 mV, cell bathed in a high K medium (see methods). Example sections of recordings made in absence and presence of propofol 100 μM. Main conductance states are indicated by dotted lines as follows C (closed), O_1_ (single channel opening), O_2_ (two channels open). B. An all-points histogram of conductance levels measured over 20 s period in presence and absence of propofol. Note peak in current at approximately ∼2.5 pA. C. Quantitative analysis of NP_open_ (the probability of channel opening) showed no significant effect of propofol on channel activity (0.2 ± 0.05 vs 0.23 ± 0.07, mean ± SEM, n = 9, p = 0.28).

Since TASK channels appear to be an important target for the effects of inhalational anaesthetics upon hypoxia sensing (Buckler et al., 2000) we investigated the effects of propofol on TASK channel activity in cell attached patches from type-1 cells. There was no significant effect of propofol 100 μM on TASK channel open probability (NP_open_, 0.19 ± 0.04 vs 0.22 ± 0.05, n = 11, p = 0.088). Fig. 8A shows the characteristic all-points histogram of the native TASK current, with an example trace in Fig. 8B. Overlaid is the histogram after the application of propofol, and it can be clearly seen that there was no major change in channel activity at any level. The peak level of channel activity in this patch was at ∼2.5 pA (Fig. 8C) which corresponds to that expected of TASK1/TASK3 heterodimers (Turner and Buckler, 2013).

### 3.6 Effect of propofol on glomus cell response to high [K^+^]o stimulation

**Fig. 9:**
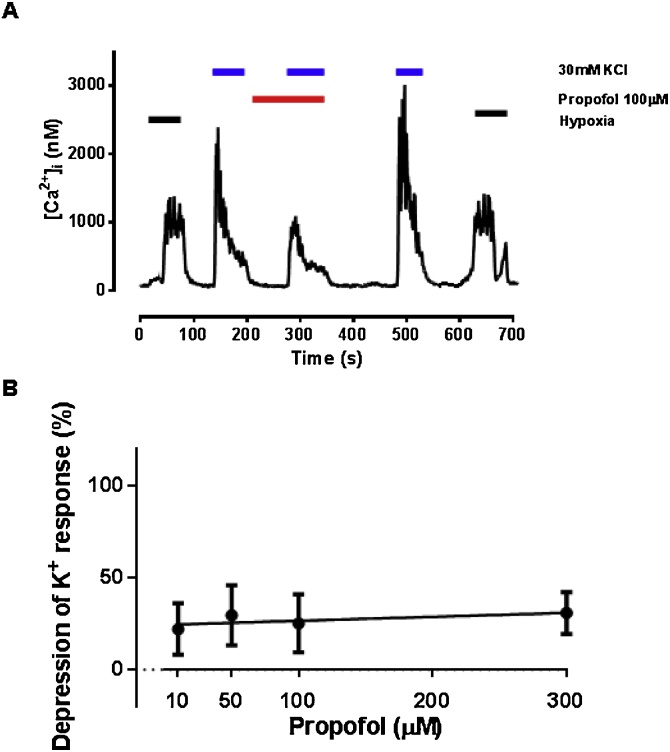
Influence of propofol on high [K^+^]_o_ induce [Ca^2+^]_i_ response in isolated glomus cells. A. Example recording of [Ca^2+^]_i_ showing response to 30 mM KCl and the partial inhibition of this response by 100 μM propofol. Note the preliminary exposure to hypoxia confirming this is an oxygen-sensitive glomus cell and the post-exposure control response to KCl and hypoxia that confirm reversibility of effects of propofol B. concentration-response relationship for effects of propofol on the [K^+^]o induced [Ca^2+^]-response. Data are expressed as a percentage depression (mean ± SEM) relative to a control (no propofol) response. All averaged data from cells exhibiting reversibility of effects of propofol.

In order to establish whether propofol inhibits voltage-gated calcium entry, or other aspects of calcium signalling, we studied its effects on the calcium response to membrane depolarisation by elevation of extracellular potassium. Propofol (100 μM) blunted the [Ca^2+^]_i_ response to 30 mM KCl (e.g., 820 ± 117 nM vs 509 ± 76 nM; n = 24, p < 0.001; Fig. 9). This effect was evident and seemingly constant/maximal across a wide range of doses from 10 to 300 μM (Fig. 9B; ANOVA for effect of dose, p = 0.775; NS).

## 4 Discussion

The main finding of this study is that propofol is capable of exerting a direct inhibitory effect on the glomus cell response to hypoxia and CO_2_, substantially diminishing the rise in [Ca^2+^]_i_ evoked by these stimuli.

### 4.1 Propofol and GABA signalling

Many actions of propofol, such as hypnotic, are thought to occur via GABA_A_ receptors (Franks, 2015). We did not however observe any effect of GABA or selective GABA agonists/antagonists on the glomus cell [Ca^2+^]_i_ response to hypoxia (Fig. 3, Fig. 4 and Table 1). Equally we did not observe any interaction between GABA and propofol in hypoxia (Fig. 3). These results are surprising since GABA_B_ receptors are thought to be present in the glomus cell and to act in a negative feedback manner to modulate TASK channel activity (Fearon et al., 2003a, 2003b). There is a theory that GABA is released from glomus cells and acts not only postsynaptically (GABA_A_) to reduce nerve excitability (Zhang et al., 2009), but also presynaptically (GABA_B_) to prevent further GABA release. Our results would suggest that any presynaptic action is weak. The fact that we did not see any effects of these drugs, or effects of combinations of GABA with propofol (Fig. 3) robustly excludes GABA signalling as responsible for propofol’s chemodepressant actions in isolated cells. We cannot however with our experimental model exclude postsynaptic actions of GABA and propofol.

### 4.2 Nicotinergic signalling role in glomus cell hypoxic response

We confirmed that nicotine, like hypoxia, stimulates a rise in [Ca^2+^]_i_ in glomus cells (Dasso et al., 1997). This was fully inhibited by propofol (Fig. 5) adding further support to the hypothesis that propofol is able to inhibit some nicotinic receptors, as is already well established (Jonsson Fagerlund et al., 2016. Notably however, the cholinergic antagonists vecuronium and MLA inhibited only the nicotine-induced, but not the hypoxia-induced rise in [Ca^2+^]_i_ (Fig. 6, Fig. 7). This suggests that nicotinic signalling mechanisms are not integral to the hypoxic response seen in *isolated* glomus cells.

These results appear to contrast with those of Jonsson, et al. (2005) and Igarashi et al. (2002) who found that vecuronium depresses hypoxia-induced activation of afferent nerves in *intact* adult rat whole carotid body preparations. These effects of neuromuscular blockers probably require the explanation that acetylcholine is released at the synaptic level which then stimulates the glomus cell and/or the afferent nerve ending. Of course such signalling mechanisms will not be present in an isolated cell preparation, but our data nonetheless demonstrate that propofol clearly has the potential to modulate chemoreceptor response by interfering with paracrine/neurocrine cholinergic neurotransmitter signalling to the glomus cell. It remains to be seen whether nicotinic receptors on the afferent nerve ending are equally propofol sensitive.

### 4.3 Propofol and TASK channels

Our result that propofol does not influence the function of TASK channels in type-1 cells (Fig. 8), which are predominantly TASK-1/TASK-3 heterodimers (Kim et al., 2009; Turner and Buckler, 2013), is consistent with previous studies using TASK-1channels expressed in oocytes (Putzke et al., 2007), and extends that work to native heterodimeric channels. Thus propofol acts to depress hypoxia- and CO_2_-sensing in a manner distinct from volatile anesthetics which increase the activity/open probability of TASK channels (Patel et al., 1999; Shin and Winegar, 2003; Sirois et al., 2000; Pandit et al., 2010a,b).

### 4.4 Propofol and Ca^2+^-signalling

We noted that propofol had a modest depressive action on Ca^2+^entry into glomus cells evoked by high [K^+^]_o_ and may therefore act in part via inhibition of Ca^2+^_v_ channels (Fig. 9). This cannot fully account for the effects of propofol on the hypoxic response since the effects of propofol on high K^+^ evoked Ca^2+^ entry are relatively limited (to only ∼25% inhibition at 300 μM propofol). By contrast at this same dose, propofol inhibits hypoxic response by ∼70% (and almost completely inhibits the response to hypoxia at 500 μM). It should be noted however that whilst there may be more than one site of action for propofol, those that only come into play at very high concentrations may be of limited clinical relevance.

With respect to possible actions of propofol on Ca^2+^_v_ channels a number of Ca^2+^_v_ channels are reported to be invulnerable to propofol (Hall et al., 1994; Orestes and Todorovic, 2010). An exception is the slow inactivating T-type Ca^2+^_v_ channel, which is blocked by propofol (Joksovic et al., 2005). Intriguingly T-type Ca^2+^_V_ channels are thought to be important in mediating the calcium response to hypoxia (Makarenko et al., 2015).

### 4.5 Summary of possible mechanisms of action of propofol

Taking all our results and those of other aforementioned groups together, it is clear that like so many anesthetics, propofol potentially has diverse actions. These include presynaptic inhibition of voltage-gated Ca^2+^-channels and depression of nicotinic excitation at the glomus cell; and postsynaptic effects including depression of nicotinic excitation and possibly augmentation of GABA-ergic inhibition at the nerve ending. There are also a number of other possibilities which we did not explore such as inhibition of phosphorylation- mediated channel regulation through Ca^2+^/calmodulin-dependent protein kinase IIm (Cui et al., 2009).

### 4.6 Potential limitations of the study

There are some self-evident limitations to our study in that it is based upon an *in vitro* preparation from an animal model (neonatal rat). In considering the possible relevance to *in vivo* situations in humans there are therefore the usual concerns with respect to species and age variations, and loss of paracrine/neurocrine/autocrine interactions. Animal studies are however the only viable approach when using invasive techniques. We, like many other research groups, employ neonatal rat tissue because of the ease of isolating glomus cells with minimal exposure to proteolytic enzymes. Much higher concentrations of enzymes are generally required for cell dissociation in adult animals. Whilst chemoreceptor function may be immature in new born animals maturational differences in the hypoxic response in rat carotid bodies largely disappear by 10–15 days (Donnelly and Kholwadwala, 1992). Despite these considerations there is a concordance of results obtained with respect to anaesthetic sensitivity of carotid body responses between rats (neonatal and adult) and human ventilatory responses, which is striking (Karanovic et al., 2010; Pandit, 2014).

We did not assess the role of propofol across a range of chemostimuli strengths. This could potentially reveal differing sensitivity to propofol at “milder” levels of hypoxia. Recent evidence suggests that differing levels of depolarisation can recruit different types of ion channel (Kang et al., 2014; Wang and Kim, 2018). If different channels are involved in Ca^2+^ signalling depending on stimulus intensity, and if anesthetic agents like propofol can act on a range of molecular targets, then results may be influenced by both stimulus intensity and agent.

### 4.7 Conclusions

In summary, we have established that propofol is capable of depressing hypoxia- and hypercapnia-induced Ca^2+^ influx into glomus cells. The mechanisms of this depression were not fully identified but are probably partly due to inhibition of voltage-gated Ca^2+^-channels. We also observed that propofol was an effective antagonist of glomus cell nicotinic receptors and so could interfere with neurotransmission/modulation at a presynaptic and/or postsynaptic level. This latter observation suggests that in a clinical setting propofol and residual neuromuscular blockade could synergise in the operative/postoperative period. There are therefore a range of mechanisms by which surgical anaesthesia could depress the hypoxic and/or hypercapnic ventilatory response through effects upon peripheral chemoreceptors. Future research is clearly needed however to fully characterise/identify novel targets of propofol and to define their clinical relevance.
